# Flexible Colloidal Molecules with Directional Bonds and Controlled Flexibility

**DOI:** 10.1021/acsnano.3c00751

**Published:** 2023-06-26

**Authors:** Yogesh Shelke, Fabrizio Camerin, Susana Marín-Aguilar, Ruben W. Verweij, Marjolein Dijkstra, Daniela J. Kraft

**Affiliations:** † Soft Matter Physics, Huygens-Kamerlingh Onnes Laboratory, Leiden University, PO Box 9504, Leiden 2300 RA, The Netherlands.; ‡ Soft Condensed Matter & Biophysics, Debye Institute for Nanomaterials Science, Utrecht University, Princetonplein 1, Utrecht 3584 CC, The Netherlands.

**Keywords:** self-assembly, confined motion, multivalent bonds, anisotropic shape, Monte Carlo (MC) simulations

## Abstract

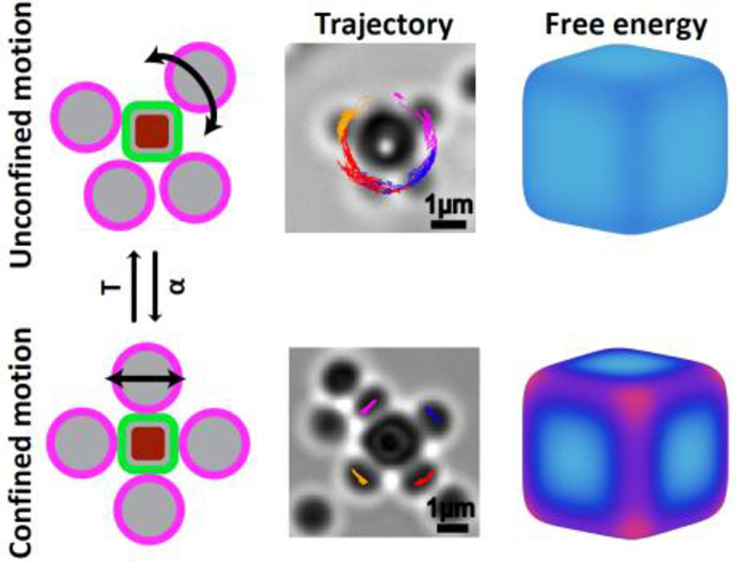

Colloidal molecules are ideal model systems for mimicking real molecules and can serve as versatile building blocks for the bottom-up self-assembly of flexible and smart materials. While most colloidal molecules are rigid objects, the development of colloidal joints has made it possible to endow them with conformational flexibility. However, their unrestricted range of motion does not capture the limited movement and bond directionality that is instead typical of real molecules. In this work, we create flexible colloidal molecules with an *in situ* controllable motion range and bond directionality by assembling spherical particles onto cubes functionalized with complementary surface-mobile DNA. By varying the sphere-to-cube size ratio, we obtain colloidal molecules with different coordination numbers and find that they feature a constrained range of motion above a critical size ratio. Using theory and simulations, we show that the particle shape together with the multivalent bonds creates an effective free-energy landscape for the motion of the sphere on the surface of the cube. We quantify the confinement of the spheres on the surface of the cube and the probability to change facet. We find that temperature can be used as an extra control parameter to switch *in situ* between full and constrained flexibility. These flexible colloidal molecules with a temperature switching motion range can be used to investigate the effect of directional yet flexible bonds in determining their self-assembly and phase behavior, and may be employed as constructional units in microrobotics and smart materials.

Colloidal molecules are excellent models for real molecules and can be used to study the influence of shape and bond directionality in self-assembly processes, phase behavior, and the creation of colloidal materials.^1−8^ However, current colloidal molecules are often rigid objects, while the functionality of many molecules such as polymers, intrinsically disordered proteins, and tRNA hinges on their ability to adapt to their structure. This conformational flexibility enables lock-and-key interactions, as well as faster and more specific binding, and has been proposed to affect their diffusive motion.^9−12^

The development of colloidal joints has recently made the fabrication of flexible colloidal molecules through self-assembly possible. These consist of solid particles or liquid droplets that can bind other particles such that they can still laterally move on their surface .^13−17^ Their intrinsic bond flexibility has been shown to enhance yields in self-assembly.^18^ Furthermore, flexible colloidal molecules have been used to demonstrate that flexibility enhances diffusion^19^ and that flexible linear and ring-like structures follow Flory theory for polymers.^15,20,21^ Besides, they have great potential for fundamental studies of their phase behavior, to understand and fabricate reconfigurable materials^22,23^ and store information.^24^

However, current realizations of flexible colloidal molecules exclusively feature bonds with an unrestricted range of motion, whereas the bonds of real molecules are often constrained to a specific range of motion due to the orbitals underlying their intramolecular bonds. The restricted motion range thus provides molecules with bond directionality. These features have not been realized in colloidal molecules yet but would be powerful not only in view of their ability to serve as model systems but also to create reconfigurable materials and functional devices with multiple stable configurations. So far, only a combination of gravitational confinement and steric hindrance of the bound particles could keep their positional order unchanged, but could not control their motion.^18^

Here, we experimentally realize flexible colloidal molecules with controlled flexibility and directionality by exploiting solid colloidal joints with an anisotropic particle shape. We demonstrate the creation of flexible colloidal molecules with directional bonds and controlled flexibility by mixing colloidal cubes equipped with surface mobile DNA-linkers^13,14,16^ with an excess number of spheres functionalized with complementary strands. The assembled colloidal molecules consist of cubes surrounded by spheres connected with multivalent bonds of DNA linkers. The anisotropic particle shape is the key ingredient, because it guides the position and controls the number of attached particles. Furthermore, the cubic particle shape constrains the lateral motion of the attached particles due to an interplay between the spatially extended multivalent bond formed between the particles and the curvature of the cube, thereby realizing a controlled flexibility. We show both by experiments and simulations that the combination of the cubic shape and DNA-mediated bonding provides confinement of the outer particles above a critical size ratio of the sphere-to-cube diameter. We justify the observed behavior by calculating the free energy of the spheres experienced at different locations on the surface of the cube based on a theoretical framework that accounts microscopically for the bond formation of surface mobile DNA linkers .^25,26^

The sensitivity of the DNA-based bonds to temperature allows us to lift confinement of the spheres to the cube sides *in situ* by a simple elevation of temperature. Small colloidal molecules with controlled flexibility and directionality can be separated using a magnet due to the permanent magnetic dipole moment of the hematite cube, providing an easy and efficient means for their exploitation. The features of bond directionality and temperature-controlled switch from limited to full flexibility make these colloidal molecules excellent model systems for studying the phase behavior of molecules and building blocks. This would also enable the assembly of reconfigurable structures with multiple stable configurations, a key ingredient for creating functional devices and machines.

## Results and Discussion

### Assembly of Colloidal Molecules with Controlled Flexibility and Directionality

**Figure 1:**
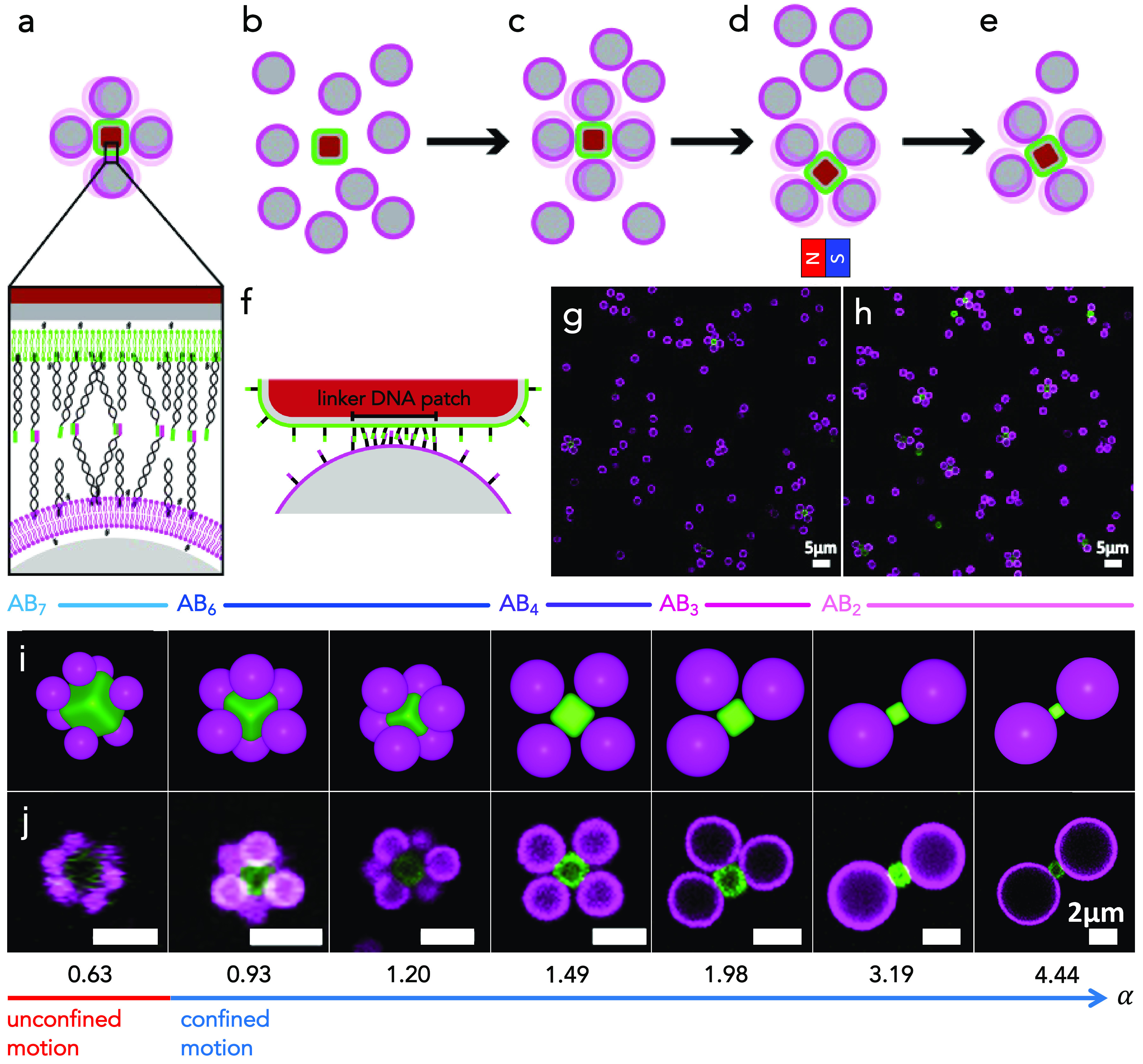
Self-assembly of colloidal molecules with controlled flexibility and bond directionality. (a) Schematic of a flexible colloidal molecule consisting of spheres (magenta) assembled onto a cube (green) and close-up view showing their functionalization with a lipid bilayer and surface-mobile DNA strands, where colored ends indicate different linker sequences. (b) The functionalized cubes and spheres are combined at a ratio of 1:20, which (c) enables rapid saturation of the cubic particles and ensures the formation of colloidal molecules. (d) The permanent magnetic dipole moment of the cubic particles is used to isolate the colloidal molecules by placing a magnet near the sample and removing the excess spheres, leading to (e) isolated flexible colloidal molecules. (f) Schematic indicating the patch formed between DNA linkers. (g, h) Representative confocal microscopy images of assembled flexible colloidal molecules prior to and following isolation. (i) Schematics of the AB_*N*_ assembled clusters and (j) representative confocal microscopy images of the most likely type of experimentally obtained colloidal molecules for varying sphere-to-cube size ratios α = 0.63, 0.93, 1.20, 1.49, 1.98, 3.19, and 4.44 (from left to right). Corresponding movies are presented in Movies S1–S7. For α = 0.63, the spheres move indistinctly on different faces of the rounded cube, while an increasingly confined motion on single faces occurs for higher α.

We created colloidal molecules with controlled flexibility and directionality by assembling spherical silica particles onto the surface of cubic colloids; see Figure 1. We used solid silica spheres in combination with rounded cubic particles (whose shape can be described as superballs^27^) made of hematite^28^ and coated with a thin silica layer by a Stöber procedure.^29^

To achieve flexible bonds, we exploited a method previously developed by some of us with modifications:^13,14,16^ the particles were functionalized with a lipid bilayer that contained dsDNA strands with and without a 11 bp single stranded end that act as linkers and steric stabilizers, respectively, see Figure 1a. Since colloids coated with mobile linker DNA are able to bind more quickly to surfaces with higher receptor density,^30,31^ we here employed 300 linker DNA strands/μm^2^ on the spherical and 20000 linker DNA strands/μm^2^ on the cubic surfaces that act as complementary linkers. In addition, we added inert DNA strands at a nominal concentration of 1× 10^5^ linkers/μm^2^ to both particles. After being mixed, the complementary functionalized cubes and spheres bind to each other by accumulating DNA linkers in the area of closest contact. In this way, a so-called DNA *patch area* is formed, as shown in Figure 1f. We do not expect all added DNA strands to be included in the lipid bilayer. However, we used these nominal concentrations because they were empirically found to balance the mobility with high binding probability. The low concentration of DNA strands on the spheres limits the number of bound DNA linkers and in this way ensures mobility of the colloids after binding.^14^ The high linker concentration on the cubes provides fast binding of the spheres, even for the consecutive adsorption of spheres, because linker accumulation in the bond area does not lead to significant depletion of linkers on the cube surface.^30,32^ In the Methods section, we report further experimental details and the DNA sequences employed.

In a typical experiment, we mixed the DNA-functionalized cubes with an excess number of spheres (cube-to-sphere number ratio 1:20) and transferred them to a customized sample holder, where polyacrylamide (PAA)-coated coverslips were used as a substrate and covered on the top. During self-assembly, the excess number of spheres and the higher concentration of linker DNA on the cubes leads to faster binding of spheres onto the cubic particles and the formation of finite-size clusters, the flexible colloidal molecules, see Figure 1b, c, and g. After 12 h, the sample holder contained clusters and unbound spheres at the bottom. To remove excess spheres, we utilized the magnetic property of the hematite cube and isolated the flexible colloidal molecules by a hand-held magnet. We did so by dispersing, separating, and redispersing the colloidal molecules in buffer twice (200 mM NaCl, 10 mM HEPES, pH 7.4) (Figure 1d), which led to a significantly increased purity and concentration of flexible colloidal molecules, see Figure 1h.

The cubic shape of the central particle allows for precise control over the maximum and most probable number of bound spheres by acting as a guiding template. The resulting colloidal molecules are of type AB_*N*_, where A indicates the cube, B the spheres, and *N* the coordination number. We employed different size ratios of the sphere diameter σ_s_ to cube edge length σ_c_, α = σ_s_/σ_c_ = 0.63, 0.93, 1.20, 1.49, 1.98, 3.19, and 4.44. The size of the cubic particles cannot be varied greatly, and hence different size ratios are achieved by varying the size of the spheres. In this way, we find different values of the number of spheres attached to the cube, with more than six spheres attached for the lowest size ratios and with at maximum one sphere per facet for α ≥ 1.20. Schematic representations and confocal microscopy images of the resulting flexible colloidal molecules are shown in Figure 1i and j, respectively, for the studied size ratios. We note that magnetic separation works well for size ratios in the range 0.63 ≤ α ≤ 1.49, but for larger size ratios from 1.98 to 4.44, we found that the increased size of the sphere caused the bond between the sphere and cube to break easily during separation.

The most probable coordination number of the colloidal molecules is the result of two factors. First, it is based on packing considerations, according to which each bounded sphere limits the available space for others to bind depending on its excluded volume.^18,33^ This effect has been extensively explored to increase the yield and sharpen the cluster size distribution for flexible colloidal molecules made of spheres only,^18^ for electrostatically assembled rigid colloidal molecules made from spheres and cubes,^34^ and for polyhedra in confinement.^35,36^ Here, no external confinement is present, which could affect the guiding effect of the cubic shape on the position of the spherical particles. Also, the anisotropic cubic surface could lead to a nonconformal distribution of the linkers that could cause an effectively different shape, as was previously shown for nanoparticles.^37,38^ However, for the colloidal particles employed here an eventual nonuniform distribution of much smaller linkers will not significantly affect their shape. For the electrostatic assembly of cubes and spheres, colloidal molecules with coordination number 6, AB_6_, were found for sphere-to-cube size ratio α < 2, while AB_4_ and AB_2_ type colloidal molecules were predominantly observed at α ≈ 3 and for α > 3, respectively. For the present case, the cubic particle at the center provides a template that guides the position of the spheres. Indeed, for α ≥ 1.20 we find approximately the same coordination numbers, as evidenced by the most likely cluster shown in Figure 1i and j. Differently from rigid colloidal molecules, we find that longer times are typically required for establishing the flexible bonds between DNA linkers, and hence we allowed assembly to continue for 12 h instead of the 15 min that are typically required for rigid colloidal molecules.^34^ We also found that employing a high number of DNA linkers on the cubic particles together with low DNA concentrations on the spheres increases the yield of colloidal molecules with a maximum valence. The reason is that this combination retains mobility by limiting the number of bound linkers through the low concentration of linkers on the spheres. At the same time, it enables maximization of the number of bound particles on the cube because it avoids depletion of linkers on the cubes upon binding of the spheres. The experimental cluster size distribution for the analyzed size ratios is reported in Figure S5.

The coordination number of the colloidal molecules is also influenced by the gravitational height which effectively confines larger spheres to quasi-2D, see Table S3. For low α = 0.63, 0.93, and 1.20, we can employ small spheres which diffuse in three dimensions. Therefore, assembly occurs mainly under three-dimensional conditions and yields predominantly AB_6_-type colloidal molecules. For higher size ratios, which imply the use of larger spheres, we mainly retrieve two-dimensional constructs, as the spheres quickly settle to the bottom of the container and assembly occurs under quasi-2D conditions. Hence, as at most only one sphere binds per facet, AB_4_ and AB_3_ colloidal molecules are obtained at α = 1.49 and 1.98, respectively. Further increasing the size ratio above α = 3.19 forces the spheres to bind to opposite sides of the cube due to steric constraints, forming mostly AB_2_ colloidal molecules.

By employing surface-mobile DNA strands, the spheres of the fabricated colloidal molecules are able to diffuse on the faces of the cube. In particular, we observed that for the smallest size ratio the spheres can move between different facets whereas the other cases, the motion is limited to the same facet of adsorption (see Movies S1–S7). In the following sections, we will first theoretically explore the reasons behind this behavior and then conduct a comprehensive analysis of the conformational flexibility of the assembled colloidal molecules.

### Origin of Constrained and Unconstrained Motion of Flexible Colloidal Molecules

The cubic shape, in combination with the multivalent bonds, plays a crucial role in enabling the spatially constrained motion of the adhered spheres and the conformational flexibility of the colloidal molecules. As previously mentioned, the bond between the spheres and cubes is composed of numerous bound DNA linkers within a patch area (Figure 1f). The size and shape of this patch are determined by two factors: (i) the distance at which two DNA linkers can still bind and ii) the shape, dimension, and relative position of the two bound particles. The motion of the spheres on the surface of the cube requires rearrangement and shrinking of the DNA patch at the edges and corners in comparison to the face.^14^ As a result, all of the strands comprising the patch will adjust their positions in the lipid bilayer with respect to their original ones. In cases in which the energy required for this rearrangement exceeds the thermal energy, crossing around a corner or edge of the cube may occur less frequently.

**Figure 2:**
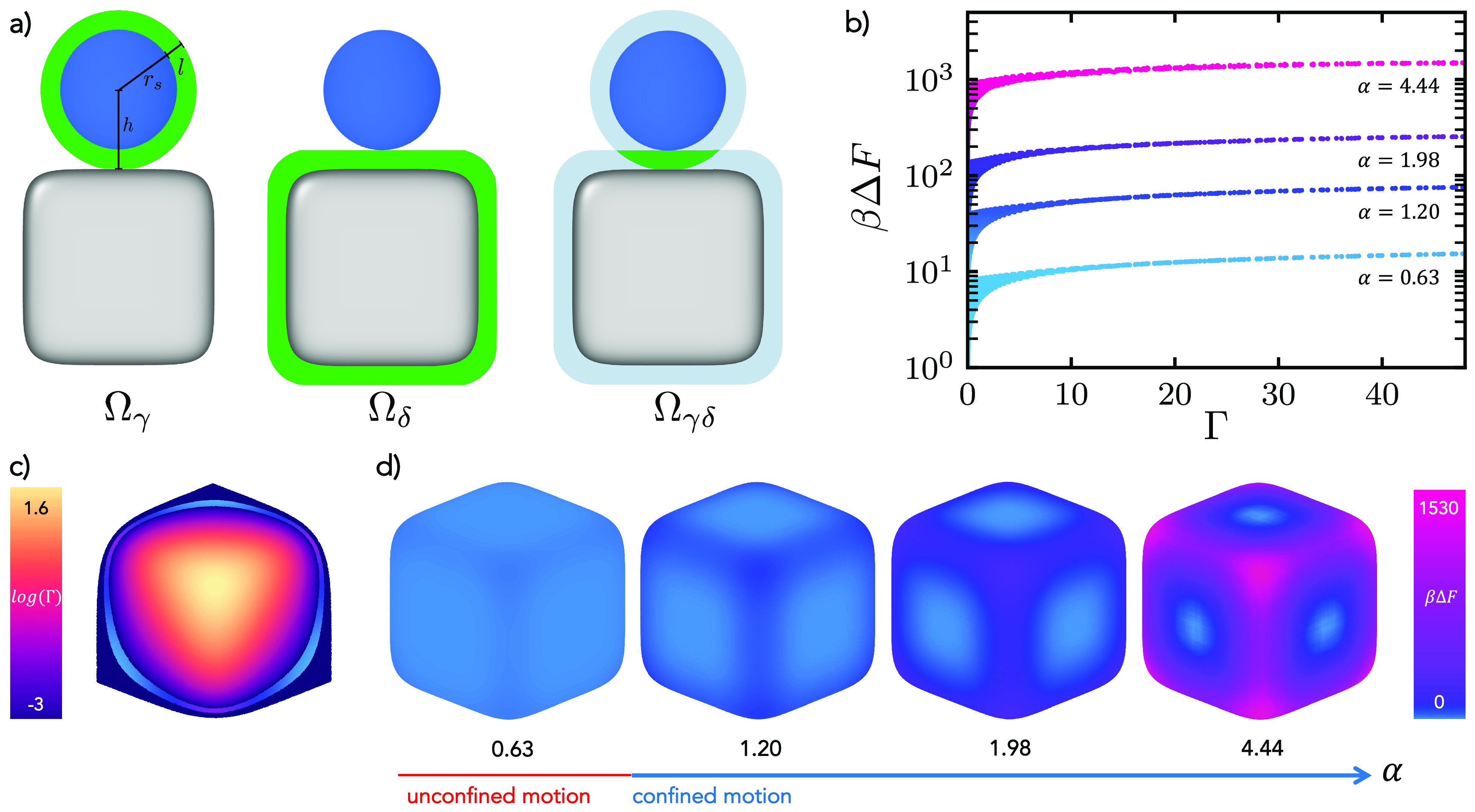
Origin of constrained and unconstrained motion. (a) Schematics showing the configurational space Ω_γ_, Ω_δ_, and Ω_*γδ*_ available for mobile strands when these are located on the spherical colloids, on the cube, and for hybridized strands, respectively. Sphere and cube are located at a distance *h* = σ_s_/2 + *l* = *r*_s_ + *l*, with *l* being the length of a DNA linker and *r*_s_ the radius of the sphere. The green color indicates the available configurational space for the strands in each case, while light blue in Ω_*γδ*_ represents the shell of thickness *l* where the strands are able to move in when unhybridized. (b) Free energy barrier βΔ*F* as a function of the Gaussian curvature Γ of the cube which is parametrized as a superball for the four different size ratios analyzed, namely, α = 0.63, 1.20, 1.98, and 4.44. (c) Gaussian curvature depicted on an octant of the superball. For a band of the octant with (similar) low curvature, we also report the corresponding free energy. For the other octants, the free-energy landscape is the same due to the symmetry of the superball. The color scale for the free energy in the band is the same as in d. (d) Free energy barrier βΔ*F* reported on the surface of the superballs for the four different size ratios analyzed in b.

To identify the nature of the constrained and unconstrained motions for different sphere-to-cube size ratios, we calculated the free energy experienced by the spheres on the surface of the cube when bonding between DNA strands occurs. To this end, we adapted a microscopic model that retains all the essential features of the physics of DNA-mediated interactions, including their mobility on the surface, to the geometry of our system,^25,39^. This model was used to describe various self-assembly processes of colloids with a fixed strand position on their surfaces,^39−42^ and then extended to account for mobile linkers .^17,25,30,43−48^ In the initial step, we compute the free energy βΔ*G*_*γδ*_ of a bond between two DNA strands γ and δ, given by
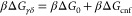
1where β = 1/*k*_B_*T* with *k*_B_ the Boltzmann constant and *T* the temperature. The first term, Δ*G*_0_, refers to the DNA hybridization free energy, while the second term, Δ*G*_cnf_, denotes the configurational cost linked to bond formation. Δ*G*_0_ is dependent on the DNA sequence employed in the strands and varies with temperature. We calculate its value using the nearest-neighbor SantaLucia rules^49^ with an inert tail correction,^50^ see the Supporting Information. Once two strands are hybridized, their available configurational space is reduced as compared to two unhybridized strands,^41,51,52^ and this is captured by:^25^
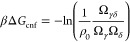
2where ρ_0_ is the reference concentration, Ω_γ(δ)_ is the configurational space available for the strand γ(δ) when unhybridized on the sphere (cube), and Ω_*γδ*_ is the configurational space for a pair of hybridized strands. The configurational space of unbound linkers consists of the volume of a shell around the surface to which the DNA linkers are attached with a width equal to the length of the linker *l*.^25^ Instead, hybridized strands will be able to explore only the region where the configurational spaces of the sphere and cube overlap; see schematics in Figure 2a and Figure S2. We use Monte Carlo integration to estimate the available configurational spaces for different positions of the sphere on top of the cube. To simplify the theoretical treatment, we set the normal distance between the center of the sphere and the surface of the cube to *h* = *l* + σ_*s*_/2 = *l* + *ασ*_*c*_/2. This allows us to neglect the repulsive contributions arising from inert and reactive DNA strands on the lipid bilayers. Further details on these calculations can be found in the Supporting Information.

We then extend the calculation of the free energy to multiple mobile strands,^25^ obtaining *βF*^bond^ (see eq S10), for which an estimate of the overall number of strands is needed. Since it is difficult to quantify the fraction of reactive and inert strands that are embedded in the lipid bilayer from experiments, we rely on the predictions of our microscopic model. In particular, we choose the number of strands as the threshold value above which the motion of the spheres for the smallest size ratio studied would be constrained, thereby contradicting experimental evidence. For higher size ratios, we then use the same strand density as that used for the smallest size ratio. We provide additional details in the Supporting Information.

In this way, we can calculate βΔ*F* = *βF*_face_^bond^ – *βF*^bond^ as the difference in free energy experienced by a sphere when it moves from the center of a cube face, where the configurational entropy is maximized (see Figure S2), to another position on its surface. To achieve this, we construct a fine discrete grid using the equation for a superball to describe the cube’s surface^27^ (see Methods), allowing us to obtain *βF*^bond^ for each point. We plot this quantity as a function of the Gaussian curvature Γ of the rounded cubes for four different size ratios α in Figure 2b. In Figure 2c, we show Γ for an octant of a cube/superball with α = 4.44 .^53,54^ The free energy βΔ*F* is also depicted directly on the surfaces of the superballs in Figure 2d. We observe that the free-energy barrier for moving across the edges increases as the diameter of the sphere increases from α = 0.63 to α = 4.44, which is consistent with our expectations. Specifically, we find that βΔ*F* increases from a few *k*_*B*_*T* for the smallest size ratio to hundreds of *k*_B_*T* for the highest size ratio. Since we assume in our calculation that Δ*G*^0^ is constant for all DNA strands, the observed increase in βΔ*F* with increasing size ratio α can be attributed to the reduced configurational entropy of the bound strands located at the edges compared to those on the faces of the superball (see also Figure S2). Consequently, the mobility of the attached sphere is impeded and the probability of crossing from one face to another decreases for larger spheres.

Additionally, we observe that for each size ratio, the largest value of the free-energy barrier is located at the highest curvature, which corresponds to the value measured at the corners of the rounded cube. This finding confirms that the probability for spheres to be primarily located at the center of the cube faces, where Ω_*γδ*_ is the highest, is enhanced. We also observe that in all cases the system exhibits an increasingly wider range of free energies as Γ approaches zero. This can be explained by examining Figure 2c, which shows both Γ and βΔ*F* for a narrow band of the superball where the curvature has approximately the same (low) values. Within this band, we notice that as we move from one face of the cube to the other we cross an edge region where the free energy varies significantly. This counterintuitive phenomenon is attributed to the differences in the configurational space that bound strands in the patch located in these two regions would experience, resulting in different free energies for sites with similar curvature. In contrast, if we had a surface with a constant curvature that was infinitely large, then the free energy would remain constant across the entire surface.

### Impact of Size Ratio on the Flexibility of the Colloidal Molecules

Using a microscopic model for the DNA interactions between the colloids, we found that the size of the spheres has a significant effect on the free energy of the system. By changing the size ratio between the spheres and the cube and thus altering the features of the DNA patch, we not only have access to different coordination numbers but also expect to have control over the conformational flexibility of colloidal molecules by adjusting the energetic barrier between different conformations. Eventually, this control will enable us to observe a transition from unconstrained motion, where spheres are able to move across various facets, to motion constrained to a single facet of the rounded cube.

**Figure 3:**
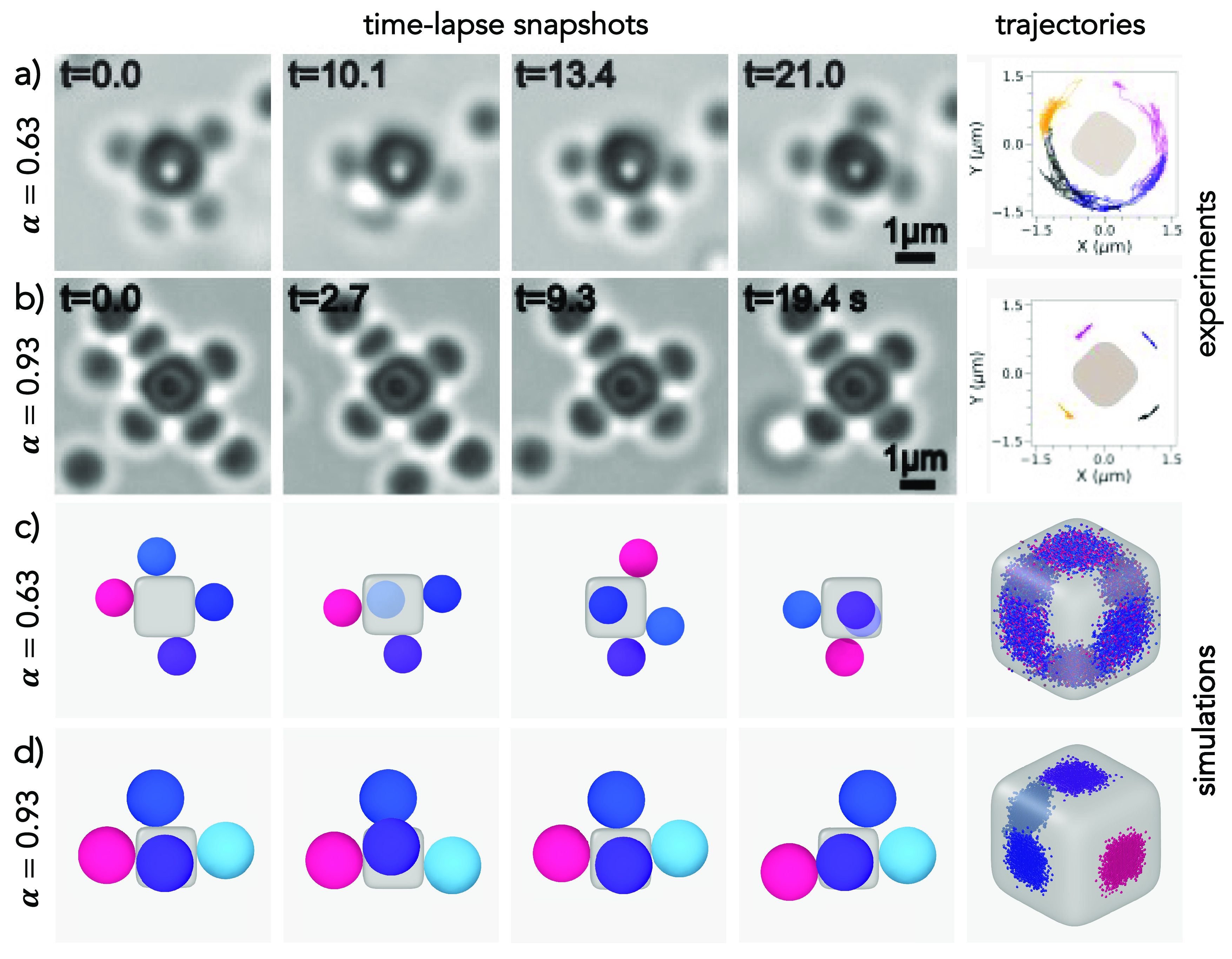
Transition from unconstrained to constrained motion. Time-lapse snapshots of microscope images showing the sphere motion on the cube for (a) α = 0.63 (unconstrained motion) and (b) α = 0.93 (constrained motion). The right panel reports the trajectories of the spheres on the surface of the cube. Corresponding movies of flexible colloidal molecules are presented in Movies S8 and S9. (c, d) Three-dimensional time-lapse simulation snapshots and trajectories for the same size ratios as in a and b, respectively. For each α, the snapshots always have a fixed orientation, and particles with the same identifier are colored alike. The rounded cube always has the same dimension in the two cases. As in a and b, the rightmost panels reports the trajectories of the sphere on the surface of the cube.

In the experiments, we thus first focus on two size ratios, namely, α = 0.63 and 0.93, and on a colloidal molecule with four spheres attached. We intentionally introduced damage to the PAA coating to (partially) immobilize the flexible colloidal molecules by adhesion to the glass slide and subsequently selected one with an immobilized cube and mobile spherical particles (see Methods). In this way, it is possible to easily track the motion of the spheres on the central cubic particle and the results for the two different size ratios are not affected by excluded volume effects. Figure 3a and b report the experimental time-lapse microscope images and the trajectories of the spheres over time for both size ratios α. The full movies are reported in the Supporting Information and Movies S8 and S9. For the smallest size ratio α = 0.63, we observed that the spheres move smoothly between the different faces of the cube, implying that they were able to cross its edges. Correspondingly, the trajectories of the attached spheres show how the spheres are able to diffuse on the surface of the cube, giving rise to an unconstrained motion. This picture drastically changes when studying α = 0.93. In this case, the motion is restricted to the facets of the cube, with the spheres always exploring the same facet where they initially bound to, as evident from the particle trajectory shown in the rightmost panel of Figure 3b. We note that the large spread in the linker distribution within the sample leads to some colloidal molecules to still show motion at a size ratio α = 0.93, while the majority displays confined motion of the spheres. Thus, the spread in the linker distribution causes a gradual transition from unconfined to confined motion with increasing size ratio.

To gain further insights, we studied the motion of spheres on the rounded cube using Monte Carlo (MC) simulations. For this purpose, we randomly place the same number of spheres as in the experiments on the surface of the cube and use the free-energy calculations presented earlier as a criterion for attempting an MC move on the surface of the cube using the same density of inert and reactive DNA strands for both size ratios. More information can be found in the Methods section and in the Supporting Information. Our simulations show that we indeed recover a behavior similar to that found in experiments. Simulation snapshots and the corresponding regions of the cube explored by the four spheres are shown in Figure 3c and d for α = 0.63 and 0.93, respectively. Such regions are made up of the corresponding contact points of the spheres onto the superball and are colored differently for different spheres. In agreement with experiments, we observe that the increase in size ratio from α = 0.63 to α = 0.93 corresponds to a transition from unconstrained to constrained motion. Indeed, we observe that small spheres with α = 0.63 change multiple times between facets, implying that their movement is free on the surface of the cube and across faces (Figure 3c).

This behavior is also reflected in the decay of the position autocorrelation function, defined as *C*_r_(*t*) = ∑_*i* = 1_^*N*^(1/*N*)(**r**_*i*_(0)**r**_*i*_(*t*))/**r**_*i*_(0)^2^, where the sum runs over all bounded particles *N* = 4, **r**_*i*_(0) and **r**_*i*_(*t*) are the initial position and the position at time *t*, respectively, of the *i*th sphere. Note that the positions are taken as the corresponding positions of the sphere on the surface of the cube. As shown in Figure S6 for α = 0.63, *C*_r_(*t*) starts already decaying after 10^4^ MC steps, implying that particles indeed diffuse freely on the surface of a cube. On the contrary, for α = 0.93, we confirm the confined movement on the initial faces of adsorption, with *C*_r_(*t*) not decaying even at long times.

**Figure 4:**
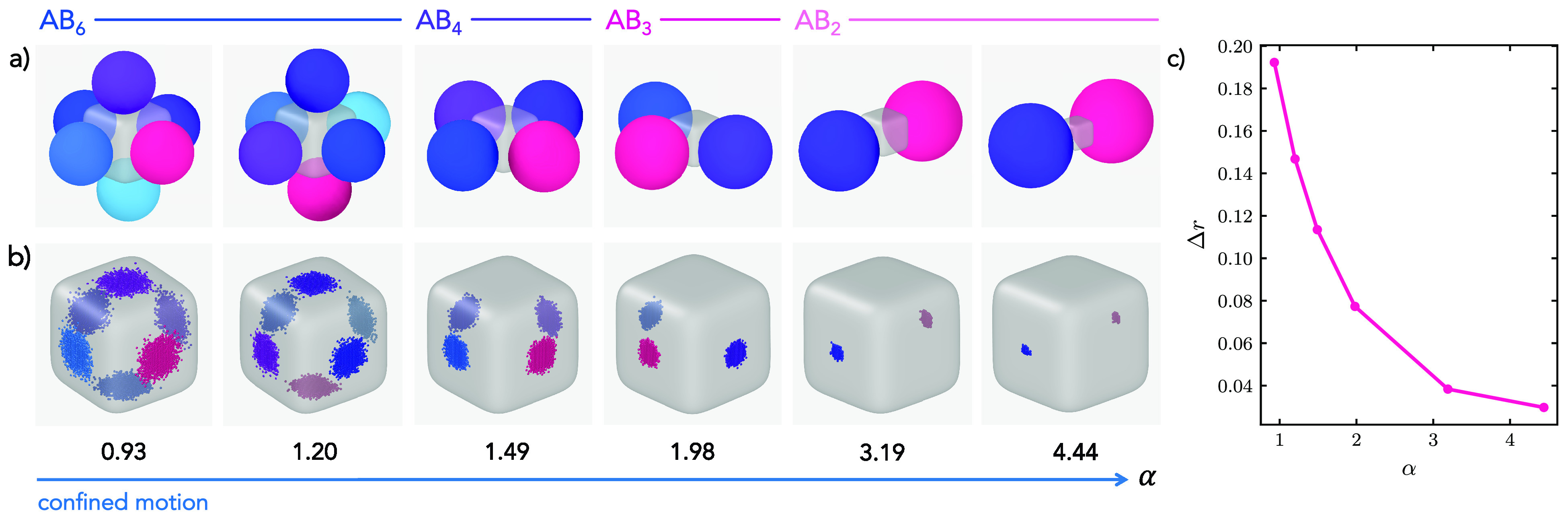
Confined motion. (a) Simulation snapshots showing an exemplary configuration of the spheres with respect to the central cube and (b) trajectories of the points of contact of the bound spheres for varying sphere-to-cube size ratios α = 0.93, 1.20, 1.49, 1.98, 3.19, and 4.44 (from left to right). For better visualization, the points of contact are reported every fifty Monte Carlo (MC) steps. Different colors are employed to distinguish different spheres. (c) Mean displacement of the spheres on the surface of the superball Δ*r* as a function of size ratio α.

The study of the dynamics of the spheres on the surface of the rounded cube can also be extended to colloidal molecules with a sphere-to-cube size ratio that exceeds 0.93. In simulations, we assess the most frequently experimentally assembled cluster for each size ratio α and we constrain the motion of the spheres in the *z*-direction to mimic the effect of the experimentally observed quasi two-dimensional confinement. Configurations adopted by such colloidal molecules and particle trajectories are shown in Figure 4a and b, respectively. The corresponding experimental movies are reported in Movies S2–S7, of which still frames are shown in Figure 1j.

Consistent with the previous observation for α = 0.93, we notice that the sphere motion is always restricted to the same facet. Nonetheless, these colloidal molecules with limited mobility of the spheres still retain a discrete amount of flexibility, which in these cases thus refers to the ability of the spheres to explore the face to which they are bound to. While all colloidal molecules indeed possess some flexibility, the range of motion of the attached spheres reduces with an increasing size ratio. This can be observed by looking at the regions explored by the spheres reported in Figure 4b, where the scattering of the sphere’s contact points is progressively reduced for higher α. This phenomenon is due to the increased patch size of the bound DNA linkers with respect to the cube size, which implies that spatial limitation by the cube facet is already experienced at smaller displacements from the center of the facet. In fact, more linkers will retain a reduced configurational entropy. Thus, with increasing α, a further decrease of the flexibility is observed. Besides, steric constraints imposed by the presence of spheres on adjacent facets of the cubes can further reduce the range of motion at higher size ratios for the same coordination number *N*. The colloidal molecules thus obtained not only differ in coordination number but also in conformational flexibility. We quantify the confinement in the motion by calculating in simulations the mean displacement of the spheres as , where we assumed the surface area in which the sphere motion occurs to be flat. We report the latter as a function of the size ratio α in Figure 4c. The progressive decrease in the size of the region explored by the spheres confirms the presence of constrained motion, which is progressively restricted toward α = 4.44 by about five times.

### Temperature Induced Reversible Transition of Flexible Colloidal Molecules from Constrained to Unconstrained Motion

The free-energy difference experienced by the spheres hinges on the DNA-based bonding patch. While the maximum configurational entropy difference between the facet and edge is determined by geometry alone, the enthalpic and entropic contributions for the formation of the DNA patch depend on the number of bound DNA linkers.

Therefore, a change in the number of bound DNA linkers in the patch tunes the free-energy landscape, providing an additional mean of controlling the conformational flexibility and eventually the confinement of the motion of the spheres. We experimentally realize this by exploiting the temperature dependent binding probability of the DNA linkers, which is controlled by the DNA hybridization free energy Δ*G*^0^. Increasing the temperature close to the melting temperature reduces the number of bound linkers and hence increases the probability of a sphere to cross to other facets.

**Figure 5:**
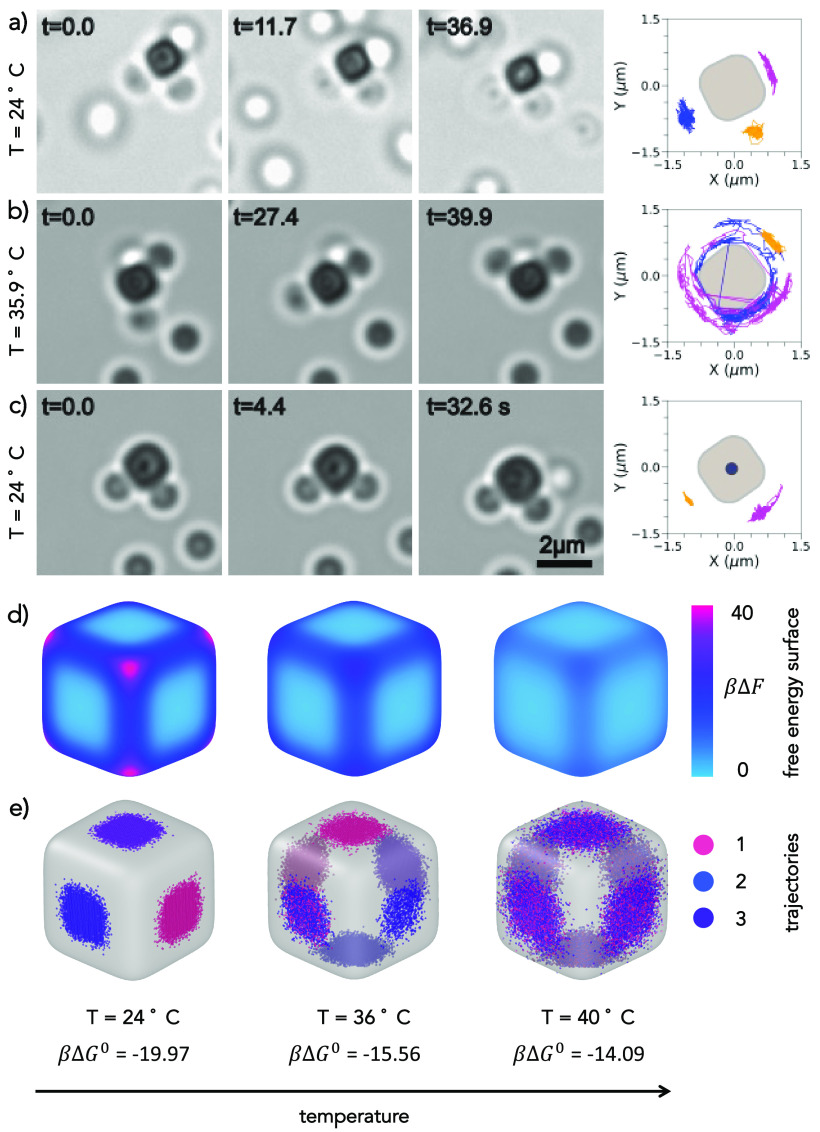
Temperature control over conformational flexibility. (a–c) Bright field microscopy snapshots and corresponding trajectories of a flexible colloidal molecule with α = 0.93 showing a reversible transition of constrained to unconstrained conformational flexibility. The cube has been immobilized on the substrate. (a) At room temperature *T* = 24 °C the diffusion of spheres is constrained to the respective faces of the cube. (b) Upon an increase in the temperature to *T* = 35.9 °C the same flexible colloidal molecule shows full conformational flexibility where spheres can cross the edges. (c) Cooling back to room temperature confines the spheres’ motion again to the respective faces of the cube. (d) Free energy βΔ*F* reported on the surface of the superballs for *T* = 24, 36, 40 °C, corresponding to three different values of the DNA hybridization free energy βΔ*G*^0^. (e) Trajectories of three spheres on the surface of the superball for the same temperatures as in (d). Different colors are related to the trajectories of different spheres.

We demonstrate the temperature dependent conformational flexibility with a flexible colloidal molecule with three bound spheres for α = 0.93. Again, we immobilize the colloidal molecules to track the particle motion (see Methods). At room temperature, the spheres are mobile but confined to their respective faces, as visible from the snapshots and trajectories of the spheres in Figure 5a. When the sample is heated to 35.9 °C, the same flexible colloidal molecule now exhibits full conformational flexibility, and the spheres bound to cubes are able to move freely from one face of the cube to another. After the sample has been brought down to room temperature, the motion of the spheres is confined once again to the individual faces of the cube, as seen from Figure 5c. The reversible change in conformational flexibility of this flexible colloidal molecule persists upon repeatedly heating and cooling the sample, as shown in Movie S10. We note that one sphere remains confined to its side for the length of the video at high temperature. This may be due to the sphere also being partially immobilized. Alternatively, it might stem from it having a higher DNA density, as the density of DNA linkers on a given sphere can vary by an order of magnitude^14,30^ and hence even at higher temperature too many bonds may persist causing its motion to be constrained. This effect is similar to the one discussed earlier, when we noted a gradual transition from unconfined to confined motion upon changing the size ratio. Between the end of the movie taken at higher temperature and the start of the next movie at lower temperature, the sphere achieved full mobility and became subsequently confined at the top of the cube. Therefore, only two traces are visible in Figure 5c.

We complement our experimental observations by investigating the effect of temperature on the conformational flexibility of the colloidal molecules via simulations (see Methods). We keep the value for the number of strands that was determined earlier for α = 0.93, and compute the DNA hybridization free energy Δ*G*^0^ at two higher temperatures, namely, *T* = 36 and 40 °C. Based on these updated free energies, we carry out new MC simulations. We do not consider any effect in polydispersity of the number of strands present in experiments since it could not be quantified precisely (see also the Supporting Information) and because its effect is mainly to make transitions from confined to unconfined less sharp upon change in size ratio or temperature.

For all cases, we report the free energy βΔ*F* and the particle trajectories on the surface of the cube in Figure 5d and e, respectively. In Figure S7, the free energies are reported as a function of the curvature of the superball. Our calculations show that, by increasing the temperature, the free-energy differences toward the edge progressively decreases. Therefore, consistent with experiments, the crossing probability for spheres increases with temperature at the size ratio analyzed. This is the reason why at the highest effective temperature studied the three particles can freely explore the entire surface of the cube, as shown in the right panel of Figure 5e. This phenomenon is similar to that observed earlier by decreasing the size ratio α between the sphere and the cube (Figures 3 and 4). However, in this case, the change in the free-energy barrier is entirely determined by the DNA hybridization free energy Δ*G*^0^, while the configurational entropy term remains constant.

To summarize, the multivalent bonding patch together with the anisotropic shape of the cube provide therefore the necessary ingredients to confine the motion of the attached spheres, and, by changing the temperature, this constraint can be reversibly relieved and imposed on demand.

## Conclusions

We assembled colloidal molecules with directional bonds and controlled the conformational flexibility by employing cubic particles at their center. By varying the size ratio α between the spheres and cubes, we assembled flexible colloidal molecules with different, well-controlled coordination numbers in high yields. We identified α, and thus the DNA patch size between the sphere and cube, to be critical in restricting the diffusive motion of the sphere to the cube’s face. At α = 0.63, we find that spheres can easily diffuse across different faces of the cube, whereas their motion is constrained to a single face for α ≥ 0.93. Using a microscopical model, we find that the curvature variation of the cube leads to an effective free-energy landscape for the spheres’ motion, with decreasing probability for crossing edges and corners with increasing size ratio. In addition, the motion on a given facet is increasingly confined due to the energetic costs associated with moving DNA linkers to the more highly curved edges. We quantified their confinement and ability to change face and analyzed their equilibrium distributions according to the free energies. In the future, it will be interesting to compare our results with new experiments that might be able to precisely quantify the number of strands incorporated into the lipid bilayer and measure their spatial arrangement on the colloid. Finally, we demonstrated that temperature can be used to reversibly switch between confined and unconfined motion of the spheres on the cubes’ surface. The magnetic dipole moment of the hematite core of the cubic particles can be utilized to separate the flexible colloidal molecules in small size ratios.

The thus prepared, flexible colloidal molecules can serve as building blocks for the preparation of higher order structures with desired flexibility and can be used to study the influence of controlled conformational flexibility on their phase behavior,^55,56^ crystal formation, and reconfiguration, which is crucial in designing materials and their properties.^57−59^ Our insights into how the geometry of a template shapes the free-energy landscape for the adhered spheres are relevant beyond an application to flexible colloidal molecules, as they can be employed to design other flexible structures with controlled conformational flexibility. The ability to release and reimpose the confinement by a simple increase and decrease of temperature is a powerful strategy to rearrange and fixate the conformation of flexible colloidal structures at will, important for creating *in situ* controllable functional devices and machines with multiple stable configurations .^60^

## Materials and Methods

### Experimental Section

#### Materials

Silica microspheres of diameters 0.97 ± 0.05 μm, 1.25 ± 0.05 μm, 1.55 ± 0.05 μm, 2.06 ± 0.05 μm, 3.32 ± 0.05 μm, and 4.62 ± 0.05 μm in 5 wt/v% suspension were purchased from Microparticles GmbH. Silica particles of diameter 0.66 ± 0.01 μm were synthesized by a Stöber method. Sodium chloride, ethanol, sodium hydroxide, ammonium hydroxide (28–30 v/v%), iron(III) chloride hexahydrate (FeCl_3_·6H_2_O), tetraethyl orthosilicate (TEOS), 4-(2-hydroxyethyl)-1-piperazineethanesulfonic acid (HEPES), and trimethoxysilyl propyl methacrylate (TPM), were purchased from Sigma-Aldrich. 1,2-Dioleoyl-*sn*-glycero-3-phosphocholine (DOPC), 1,2-dioleoyl-*sn*-glycero-3-phosphoethanolamine-N-[methoxy(polyethylene glycol)-2000] (DOPE-PEG2000), 1,2-dioleoyl-*sn*-glycero-3-phosphoethanolamine-N-(lissamine rhodamine B sulfonyl) (DOPE-Rhodamine), and dye 23-(dipyrrometheneboron difluoride)-24-norcholesterol (TopFluor-Cholesterol) were obtained from Avanti Polar Lipids, Inc. We used Milli-Q water for all experiments. DNA strands were purchased from Eurogentec. The sequences of DNA used were:

Strand A: double stearyl-HEG-5′-TT-TAT-CGC-TAC-CCT-TCG-CAC-AGT-CAC-CTT-CGC-ACA-GTC-ACA-TTC-AGA-GAG-CCC-TGT-CTA-GAG-AGC-CCT–GCC-TTA-CGA-*GTA-GAA-GTA-GG-3*′*-6FAM*
Strand B: double stearyl-HEG-5′-TT-TAT-CGC-TAC-CC-–TCG-CAC-AGT-CAC-CTT-CGC-ACA-GTC-ACA-TTC-AGA-GAG-CCC-TGT-CTA-GAG-AGC-CCT-GCC-TTA-CGA-*CCT-ACT-TCT-AC-3*′*-Cy3*
Strand C: 5′-TCG-TAA-GGC-AGG-GCT-CTC-TAG-ACA-GGG-CTC-TCT-GAA-TGT-GAC-TGT-GCG-AAG-GTG-ACT-GTG-CGA-AGG-GTA-GCG-ATT-TT-3′
Strand D: double stearyl-5TT-TAT-CGC-TAC-CCT-TCG-CAC-AGT-CAA-TCT-AGA-GAG-CCC-TGC-CTT-ACG-A
Strand E: TCG-TAA-GGC-AGG-GCT-CTC-TAG-ATT-GAC-TGT-GCG-AAG-GGT-AGC-GAT-TTT

The linker sequence in strand A and strand B is italicized.

#### Synthesis of Silica Cubes with Hematite Core

The hematite cubes of edge length 0.83 μm were synthesized following reference^28^ and coated with 0.105 μm silica layer using the process described in the literature.^29^ In a typical synthesis of hematite cubes, 100 mL of aqueous 2 M FeCl_3_·6H_2_O was prepared in a 500 mL of a Pyrex bottle. Next, 100 mL of 5 M NaOH solution were added while stirring for 20 s. Then, the mixture was stirred continuously for another 10 min and subsequently placed in a preheated oven and left undisturbed at 100 °C for 8 days. The resulting hematite cubes were washed several times using centrifugation and redispersed in MilliQ water. To coat them with a thin layer of silica, 100 mL of ethanol and 0.6 g of synthesized cubes were mixed under sonication and mechanical stirring in a 2-neck round-bottom flask at 50 °C. Subsequently, 5 mL of water, 15 mL of ammonium hydroxide solution, and tetraethyl orthosilicate (TEOS) were poured into the reaction flask. Next, the silica layer was allowed to grow on the cubes surface for 5 h. The resulting particles were first washed with ethanol and then with water to remove unreacted chemicals by repeated centrifugation and redispersion.

#### Preparation of Small Unilamellar Vesicles (SUVs)

Small unilamellar vesicles (SUVs) were prepared using a protocol described in the literature.^16^ For the preparation of SUVs, we used 77 μL of 25 g/L DOPC, 7.34 μL of 10 g/L DOPE PEG 2000, and 2 μL of either 1 g/L dye DOPE-rhodamine or 2 μL of 1 g/L TopFluor-Cholesterol dissolved in chloroform which were mixed together in a glass vial. Subsequently, the lipid mixture was dried for at least 2 h in desiccator (Kartell) attached to a vacuum pump (KNF LABOPORT N816.3KT.18). Then, 1 mL of buffer solution consisting of 50 mM NaCl and 10 mM HEPES at pH 7.4 was added to the dried lipid. The prepared solution was vortexed for 30 min during which the solution became turbid indicating the formation of giant multilamellar vesicles. The dispersion of giant multilamellar vesicles was extruded (Avanti Polar Lipids mini extruder) 21 times through a 50 nm polycarbonate membrane supported with filter paper to achieve SUV formation. The prepared SUVs were stored in the fridge at 4 °C and used for up to 3 days.

#### DNA Hybridization

We used DNA strands with a hybridized backbone, a single stranded end (linker), and inert double-stranded DNA strands for bonding and stabilizing the colloids, respectively. Prior to use, single-stranded DNA was hybridized with the complementary backbone. Strand A was hybridized with strand C to yield double-stranded linker DNA, strand B with strand C to obtain complementary double-stranded linker DNA, and strand D with strand E to create double-stranded inert DNA (DNA strands are listed in the materials section). For hybridization, we typically mixed 10 μL of 20 μM single strands and 10 μL of 20 μM complementary backbone in 90 μL of buffer (200 mM NaCl, 10 mM HEPES, at pH 7.4) solution. The DNA solutions were placed in a preheated oven at 94 °C for 30 min. The oven then was switched off and allowed to cool slowly overnight. After cooling, the hybridized DNA strands were stored at 4 °C and used for up to 2 months.

#### Functionalization of Colloidal Particles with a Lipid Bilayer Containing Linker and Inert DNA

To coat particles with a lipid bilayer, we used a 25:1 surface ratio of SUVs to particles. We maintained the same surface area ratio of SUVs to particles when coating differently sized particles. Typically, for 1 μm particles, we use 100 μL of 0.25 wt/v% particles in Milli-Q water and mixed them with 48 μm 0.5 g/L SUVs. Then, the dispersion was rotated at 8 rpm for 1 h. During this period, SUVs collide, burst, spread, and form a bilayer on the particles’ surface. Then, the coated particles were centrifuged at 800 rpm for 2–5 min, and the supernatant containing excess SUVs was removed using a micropipette. Subsequently, a concentration of 300 linker DNA strands/μm^2^ surface area was added to the spheres and 2 × 10^4^ strands/μm^2^ to the cubes. 1 × 10^5^ strands/μm^2^ of inert DNA was added to both particle suspensions. The suspensions were rotated for another 1 h. Thereafter, each suspension was centrifuged and washed 2 times with a 50 mM NaCl buffer and then once with 200 mM NaCl buffer. The final suspension was used in self-assembly experiments.

#### Sample Preparation, Magnetic Separation, and Imaging

For all self-assembly experiments, a sphere-to-cube number ratio of 20:1 was maintained. In a 1.5 mL vial, 50 μL of functionalized 1 μm spherical particles and 2.5 μL of cubic particles were combined with 500 μL of buffer 2 solution. The mixture was then transferred to a customized sample holder and allowed to self-assemble for 12 h. Polyacrylamide (PAA) coated coverslips were used as a substrate. The coverslips were coated with PAA by adapting the protocol in the literature.^19^

For magnetic separation of the colloidal molecules after assembly, the sample holder was turned upside down to distribute the particles and colloidal molecules in 3D. Subsequently, a magnet was placed at the bottom of the sample holder to attract the flexible colloidal molecules while the nonmagnetic excess spheres remained suspended in the sample. After 5 min, the supernatant containing the excess spheres was removed using a micropipet and the sample was resuspended in 200 mM NaCl buffer solution. The same procedure was repeated one more time to remove unbound spheres.

To study how the conformational flexibility of the colloidal molecules changes with temperature, self-assembled α = 0.93 flexible colloidal molecules were deposited on a coverslip with a damaged PAA coating. The PAA coating on the coverslip partially removed by scratching it with a fine needle. The sample was then placed on a custom-made microscope stage attached with heating and cooling water circulator (JULABO DYNEO DD-300F) and temperature was monitored in the sample. The sample was heated in 1 °C steps and allowed to equilibrate for 10 min for each step during the heating cycle and cooled linearly by circulating water through the microscope stage.

Images and videos were captured with a Nikon inverted TI-E microscope equipped with an A1 confocal scan head and a brightfield mode equipped with Prime BSI Express camera (Teledyne Photometrics). The images were taken using a 100× oil objective (N.A. 1.4) at frame rates of up to 25 fps.

### Numerical Section

#### System Details

The rounded cube belongs to the family of shapes known as superballs,^27^ which interpolate between spheres and cubes. The shape of the superball is described by the equation
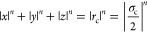
3where *n* determines the degree of roundness of the superball, while σ_c_ represents the side length of the superball, which is also used as the unit of length in our modeling and simulations. By comparing to the experimental size, we find that σ_c_ = 1.04 μm. We fix *n* = 6 to roughly capture the shape of the hematite cubes, while varying *n* from 2 to infinity produces all intermediate shapes between a sphere and a cube with sharp edges and corners.

We keep the size of the superball fixed and vary the size ratio of α = σ_s_/σ_c_ from 0.63 to 4.44. In all cases, we ensure that the magnitude of the normal vector connecting the surface of the cube to the center of the sphere is , where *l* = 0.025σ. Following refs (53 and 54) and using eq 3, we calculate the Gaussian curvature at different positions on the surface of a superball. The positions of the sphere on the superball are limited to ∼60 000 discrete grid points, for which we compute the free energy. Figure S1 illustrates a schematic of the model.

#### Monte Carlo Simulations

To study the mobility of the spheres on the surface of the superball, we perform Monte Carlo simulations based on the free energy *βF*^bond^ (Supporting Information). The free energy *βF*^bond^ is calculated by setting the number of spheres to *N*_s_ = 1, regardless of the number of bonded spheres for each size ratio. We note that varying *N*_s_ and taking *βF*_pair_ = *βF*^bond^/*N*_s_ as the energy experienced by a sphere on the surface of the superball would result in a different estimate by at most ∼4% for the highest size ratio. Therefore, for simplicity, we take *N*_s_ = 1 as the pair interaction energy for all size ratios, assuming the binding behavior of each colloid to be independent. This assumption is further supported by Figure S4, which shows that above a certain number of strands *n*_δ_ embedded on the cubic colloid, the difference in free energy experienced by a sphere at different positions on the cube does not vary. In our system, the *n*_δ_ available for binding is sufficiently high to not observe any change in the free-energy landscape when more spherical particles are bonded. A discussion on the specific choice of the number of strands for the spheres and the cubes is provided in the Supporting Information.

We initialize the system by randomly placing on the grid points of the cube the number of spheres that is observed in the experiments for a specific size ratio α. MC moves are then attempted to one of the nearest-neighbor points on the surface grid of the cube using the Metropolis algorithm, which is appropriately corrected for the number of neighbors of each surface point. We first equilibrated the system for 10^5^ MC steps before recording trajectories for at least 10^7^ MC steps. For each size ratio, we run at least 20 independent simulation runs. We also consider the effects of experimental gravitational height, where the assembly and motion of spheres are restricted in space depending on the size of the bonded spheres. For size ratios α ≥ 1.49, the motion is confined to quasi-two-dimensional conditions, which amounts to excluding the upper and lower faces of the cube. No such restriction is imposed for smaller α.

## Supporting Information Available

Parameters employed for the calculation of the free energy *βF*_bond_ for different the size ratios α. Gravitational height of the silica particles, cluster size distribution of colloidal molecules of various size ratios, schematics of the numerical model and free energy Δ*G* as a function of the Gaussian curvature of the superball Γ for the three different effective temperatures. Calculation repulsive free energy, attractive free energy for a pair of DNA strands and total free energy associated with linking a sphere and a cube. Estimate of the density of DNA strands (PDF)
Movie S1: Confocal microscopy video of a flexible colloidal molecule consisting of 0.66 ± 0.01 μm diameter spheres and a 1.04 ± 0.04 μm cube, equivalent to a size ratio α = 0.63; video is shown in real time with a frame rate of 15.2 frames per second (MP4)
Movie S2: Bright-field microscopy video of a flexible colloidal molecule consisting of 0.97 ± 0.05 μm diameter spheres and a 1.04 ± 0.04 μm cube, equivalent to a size ratio α = 0.93; video is shown in real time with a frame rate of 20 frames per second (MP4)
Movie S3: Bright-field microscopy video of a flexible colloidal molecule consisting of 1.25 ± 0.05 μm diameter spheres and a 1.04 ± 0.04 μm cube, equivalent to a size ratio α = 1.20; video is shown in real time with a frame rate of 25 frames per second .(MP4)
Movie S4: Confocal microscopy video of a flexible colloidal molecule consisting of 1.55 ± 0.05 μm diameter spheres and a 1.04 ± 0.04 μm cube, equivalent to a size ratio α = 1.49; video is shown in real time with a frame rate of 7.9 frames per second (MP4)
Movie S5: Bright-field microscopy video of a flexible colloidal molecule consisting of 2.06 ± 0.05 μm diameter spheres and a 1.04 ± 0.04 μm cube, equivalent to a size ratio α = 1.98; video is shown in real time with a frame rate of 18.3 frames per second (MP4)
Movie S6: Bright-field microscopy video of a flexible colloidal molecule consisting of 3.32 ± 0.05 μm diameter spheres and a 1.04 ± 0.04 μm cube, equivalent to a size ratio α = 3.19; video is shown in real time with a frame rate of 18.5 frames per second (MP4)
Movie S7: Confocal microscopy video of a flexible colloidal molecule consisting of 4.62 ± 0.05 μm diameter spheres and a 1.04 ± 0.04 μm cube, equivalent to a size ratio α = 4.44; video is shown in real time with a frame rate of 1.9 frames per second (MP4)
Movie S8: Bright-field microscopy video of a flexible colloidal molecule consisting of 0.66 ± 0.01 μm diameter spheres and a 1.04 ± 0.04 μm cube, equivalent to a size ratio α = 0.63; video is shown in real time with a frame rate of 16.7 frames per second (MP4)
Movie S9: Bright-field microscopy video of a flexible colloidal molecule consisting of 0.97 ± 0.05 μm diameter spheres and a 1.04 ± 0.04 μm cube, equivalent to a size ratio α = 0.93; video is shown in real time with a frame rate of 10 frames per second (MP4)
Movie S10: Bright-field microscopy video of temperature switchable motion of a flexible colloidal molecule consisting of 0.97 ± 0.05 μm diameter spheres and a 1.04 ± 0.04 μm cube, equivalent to a size ratio α = 0.93 (MP4)

## Notes

There is a preprint version of this work: Shelke, Y.; Camerin, F.; Marín-Aguilar, S.; Verweij, R.W.; Dijkstra, M.; Kraft, D.J. Flexible Colloidal Molecules with Directional Bonds and Controlled Flexibility. *arXiv*, 2023, arXiv:2301.10024.
