# Supplementary material for: Flexible Colloidal Molecules with Directional Bonds and Controlled Flexibility
Source: ACS Nano. 2023 Jun 26;17(13):12234–46. doi: 10.1021/acsnano.3c00751 (PMC10339793; doi:10.1021/acsnano.3c00751)
Supplement: Supplementary file 1 — nn3c00751_si_001.pdf [file nn3c00751_si_001.pdf]

Flexible colloidal molecules with directional bonds and controlled flexibility

Yogesh Shelke,[†] Fabrizio Camerin,[‡] Susana Marín-Aguilar,[‡] Ruben W. Verweij ,[†] Marjolein Dijkstra,[‡] and Daniela J. Kraft[*,†]

†*Soft Matter Physics, Huygens-Kamerlingh Onnes Laboratory, Leiden University, PO Box 9504, 2300 RA Leiden, The Netherlands.*

‡*Soft Condensed Matter & Biophysics, Debye Institute for Nanomaterials Science, Utrecht University, Princetonplein 1, 3584 CC Utrecht, The Netherlands.*

E-mail: kraft@physics.leidenuniv.nl

Free energy calculation

Here, we provide a detailed description of the calculation of the free energy associated with the assembly of spherical colloids on the surface of a rounded cube coated with mobile DNA strands.

System details

System details are reported in the Methods section. A schematic of the model we employ is reported in Fig. S1.

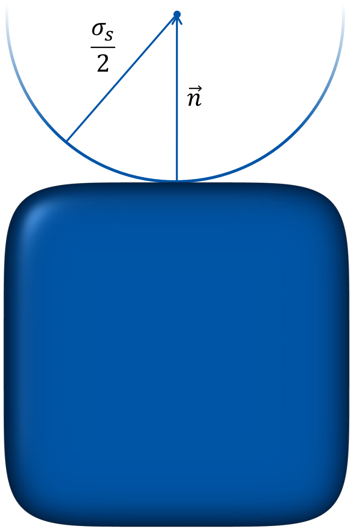

Figure S1: Schematics illustrating the numerical model, which consists of spheres of size σ_s moving on the surface of a rounded cube of size σ_c whose shape can be described as a superball. The normal vector $\vec{n}$ connects the center of the sphere to the surface of the rounded cube.

Attractive free energy for a pair of DNA strands

In this section, we describe the calculation of the attractive part of the free energy for a system consisting of a sphere and a superball. The theoretical treatment is inspired and adapted from Ref.[1] As with other models for DNA-mediated assemblies, the first step is to quantify the contributions to the free energy of *single strands* in their hybridized and unhybridized states, thus providing an estimation of the bond energy $\beta\Delta G_{\gamma\delta}$ due to the linking of two complementary strands, denoted as γ and δ. This can be expressed as:

$$\beta\Delta G_{\gamma\delta} = \beta\Delta G_0 + \beta\Delta G_{cnf}, \tag{S1}$$

where the first term ΔG_0 corresponds to the hybridization energy, which depends solely on the DNA sequence and the salt concentration in the experiments. To calculate this term, we consider the complementary ssDNA end (5'-GTAGAAGTAGG-3' and its complementary sequence), as defined in the Methods section. We calculate ΔG_0 using the nearest-neighbor (NN) SantaLucia approximation,[2] with a salt concentration correction corresponding to 200

Table S1: Contributions to ΔG_0 for three temperatures.

Temperature [°C]	NN[2] [kcal/mol]	Salt correction [kcal/mol]	Inert tail correction [kcal/mol]	ΔG_0 [kcal/mol]	ΔG_0 [$k_B T$]
24	-14.00	1.83	0.34	-11.83	-19.97
36	-11.39	1.83	0.34	-9.22	-15.56
40	-10.52	1.83	0.34	-8.35	-10.56

mM NaCl and the inert tail correction due to 77 bp of the dsDNA.[3] A summary of each contribution to ΔG_0 is given in Table S1.

The second term in Eq. S1 can be interpreted as a penalty in configurational entropy, as the hybridized strands are constrained to a smaller configurational space compared to their unhybridized state.[4–6] It can be written as

$$\beta \Delta G_{cnf} = -\ln \left(\frac{1}{\rho_0} \frac{\Omega_{\gamma\delta}}{\Omega_\gamma \Omega_\delta} \right), \tag{S2}$$

where $\rho_0 = 1\text{M} = 6.76 \times 10^8 \sigma_c^{-3}$ is a standard concentration, $\Omega_{\gamma(\delta)}$ is the configurational space available for a strand $\gamma(\delta)$ when unhybridized and located on a sphere (cube), and $\Omega_{\gamma\delta}$ is the corresponding space for a pair of hybridized strands. $\Omega_{\gamma(\delta)}$ and $\Omega_{\gamma\delta}$ have units of σ_c^3. A common approximation in this context is to treat the strands as rigid rods with an attractive tip,[7] as the dsDNA is smaller than the DNA persistence length at all temperatures investigated,[8] and the interacting ssDNA is smaller than the dsDNA. Depending on the geometry in which the strands are anchored, $\Omega_{\gamma(\delta)}$ can be calculated analytically. In the case of immobile linkers, the available volume accessible to the DNA strands is typically limited to a hemisphere around the point where the linker is attached to the surface.[4] However, for mobile linkers, the volume accessible to the DNA is enlarged due to the freedom of the strands to move on the entire surface of the colloid to which they are attached. Specifically, the DNA strands can explore the volume of a shell of width l around the surface onto which they are bound, where l is the length of the linker. The available space will differ depending

on whether the DNA is located on the sphere or on the superball surface. Furthermore, if both particles are within interacting distance, the excluded volume due to both particles must be considered.

For strands located *on the surface of a sphere* with radius $r_s = \sigma_s/2 = \alpha\sigma_c/2$, in the case that the sphere and the superball are at large distances from each other, the available configuration space Ω_γ corresponds to the volume of the spherical shell V_s^{tot}, which is obtained by taking the difference in volume between a sphere of radius $r_{s+l} = \alpha\sigma_c/2 + l$ and a sphere of radius $r_s = \alpha\sigma_c/2$ (see Figure S2(a)):

$$\Omega_\gamma = V_s^{tot} = V_s(\alpha\sigma_c/2 + l) - V_s(\alpha\sigma_c/2), \tag{S3}$$

where $V_s(r)$ is the volume of a sphere with radius r. Instead, in case the particles are at interacting distance, the volume inaccessible due to steric hindrance needs to be subtracted from V_s^{tot}. This excluded volume is shown in Figure S2(c) as the red region, while Ω_γ corresponds to the green region. Hence, we have:

$$\Omega_\gamma = V_s^{tot} - V_{over}(c, s + l), \tag{S4}$$

where $V_{over}(c, s+l)$ is the volume of overlap between a sphere of radius $r_{s+l} = \alpha\sigma_c/2 + l$ and the cube. This volume can be estimated numerically using Monte Carlo (MC) integration.

For strands anchored *on the surface of the superball*, Ω_δ is calculated in a similar way, obtaining V_c^{tot}, that is the volume of a shell of width l around the superball (see Figure S2).

The volume of a superball with radius r and power n (see Equation 3 in the main text) can be calculated either by MC integration or analytically as:[9]

$$V_c(r; n) = (2r)^3 \frac{(\Gamma(1 + 1/n))^3}{\Gamma(1 + 3/n)}, \tag{S5}$$

where Γ is the Euler-Gamma function. From this, we can directly calculate the volume of

the superball with radius $r_c = \sigma_c/2$ and power $n = 6$, $V_c(\sigma_c/2; n)$, which are the parameters used to capture the shape of the hematite cubes (see Methods). To obtain the volume of a constant shell of thickness l around such a superball, we then need the volume of a second superball with radius $r_{c+l} = \sigma_c/2 + l$ controlled by the power n', $V_c(\sigma_c/2 + l; n')$ (also estimated using Eq. S5), from which $V_c(\sigma_c/2; n)$ can be subtracted. The exponent n' is estimated numerically, yielding $n' = 5.55383$. We note that without using a superball with power n', we would get a wrong estimate of the shell volume, following a non-constant thickness around the first superball especially on the corners and the edges.

Therefore, the total volume V_c^{tot} of the shell around a superball of radius $\sigma_c/2$ and power n reads

$$\Omega_\delta = V_c^{tot} = V_c(\sigma_c/2 + l; n') - V_c(\sigma_c/2; n). \tag{S6}$$

Once more, if the sphere is at interacting distance, we should also take into account the volume inaccessible to the strands. This corresponds to the volume of overlap between a sphere of radius $r_s = \alpha\sigma_c/2$ and the superball of power n' and radius $r_{c+l} = \sigma_c/2 + l$ (see Fig. S2(c). The value of Ω_δ in this case is given by

$$\Omega_\delta = V_c^{tot} - V_{over}(c + l, s), \tag{S7}$$

where $V_{over}(c+l, s)$ is calculated via MC integration. Note that, $\Omega_{\gamma(\delta)}$ depends on the relative position of the sphere on the superball and on their reciprocal distance.

Finally, to estimate the configurational space available for the *two hybridized strands* $\Omega_{\gamma\delta}$, we follow a similar approach. We consider the sphere and superball with their corresponding shells of thickness l, and calculate their overlap region from which the inaccessible volumes due to steric effects has to be subtracted. This yields the volume that the two hybridized strands can explore, given by:

$$\Omega_{\gamma\delta} = V_{over}(c + l, s + l) - V_{over}(c, s + l) - V_{over}(c + l, s), \tag{S8}$$

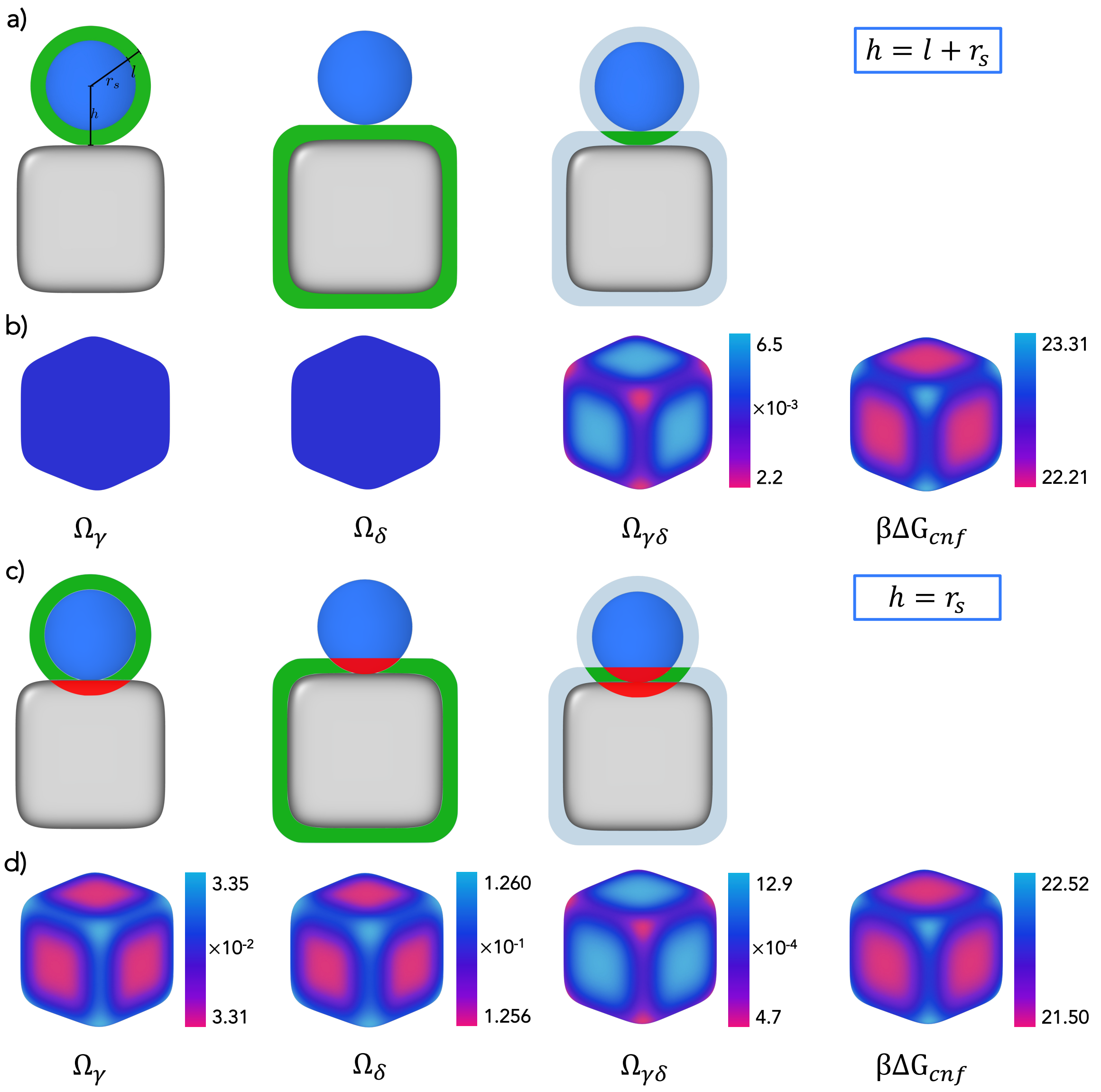

Figure S2: Schematics of the accessible configurational spaces for Ω_γ, Ω_δ and $\Omega_{\gamma\delta}$ for a distance between the center of the sphere and the surface of the cube (a) $h = l + r_s$ and (c) $h = r_s$, respectively. The green regions indicate the accessible space for strands while the red regions are removed volumes because of the excluded volumes of the colloids. The computed accessible configurational spaces Ω_γ, Ω_δ and $\Omega_{\gamma\delta}$ displayed on the surface of the superball for $\alpha = 0.63$ are reported in (b) and (d) for two respective distances h. In (b), Ω_γ and Ω_δ are constant over the whole superball and equal to 0.0337 and 0.1263, respectively. $\Omega_{\gamma\delta}$ varies on the surface of the superball and takes values as indicated in the color bar. In (d), the configurational spaces differ depending on the relative sphere-cube position both in the unbound and bound states. Configurational spaces are given in units of σ_c^3. The results presented in the main text are for $h = l + r_s$.

where the first term corresponds to the overlap of the two extended shapes, while the second and third terms represent the inaccessible volumes (see Figure S2). The green region in the

figure corresponds to $\Omega_{\gamma\delta}$, while the red regions correspond to $V_{over}(c, s+l)$ and $V_{over}(c+l, s)$.

In all our calculations we fix the distance between the surface of the cube and the center of the sphere to $h = l + r_s$ (see also below) as schematically shown in Figure S2(a). In Figure S2(b), we present the numerical estimates for the available configurational spaces $\Omega_{\gamma(\delta)}$ and $\Omega_{\gamma\delta}$ depicted on the surface of the superball for an example size ratio $\alpha = 0.63$. As expected, $\Omega_{\gamma(\delta)}$ is constant all around the surface of the two colloids since it corresponds to the volume of a shell of thickness l around the sphere (cube). On the other hand, the configurational space available for hybridized strands varies depending on the reciprocal position of the sphere and cube, and it is highest at the center of the faces of the cube. This gives rise to the overall free-energy landscape, which is reported in Figure 2 of the main text. For completeness, we summarize the schematics for the case in which $h = r_s$ in Figure S2(c). In Figure S2(d) we report the corresponding numerical estimates. We note that in this case the configurational space for the unbound strands $\Omega_{\gamma(\delta)}$ is different in different regions of the cube, with a minimum configurational entropy in the corners and edges of the cube. Nonetheless, despite a slightly different scale, the overall free-energy landscape $\beta\Delta G_{cnf}$ has similar features independently of the relative distance between sphere and cube h.

Repulsive free energy

So far, we have only considered attractive interactions between a pair of strands. However, as the colloids get closer, the strands also give rise to an effective repulsion that adds to the steric repulsion experienced by the colloids. As detailed in Ref.,[1] this contribution can be calculated as

$$\beta F_{rep} = -\ln\left(\frac{V_c^{tot} - V_{over}(c+l, s)}{V_c^{tot}}\right). \tag{S9}$$

Since we assume a constant surface distance h between the colloids, $\beta F_{rep} = 0$ as there is no overlap between a superball of radius $r_{c+l} = \sigma_c/2 + l$ and a spherical colloid of radius $\alpha\sigma_c/2$.

Total free energy

Once we have defined the individual contributions of two strands, we can calculate the *overall* free energy associated with linking a sphere and a superball in an arbitrary relative position Θ. Following the approximations made in Ref.,[4,10] the attractive free energy of a system of two particles with complementary DNA strands γ and δ can be written as:

$$\beta F^{bond}(\Theta) = n_\gamma \left[\ln p_\gamma(\Theta) + \frac{1}{2} - \frac{p_\gamma(\Theta)}{2} \right] + N_s n_\delta \left[\ln p_\delta(\Theta) + \frac{1}{2} - \frac{p_\delta(\Theta)}{2} \right], \quad \text{(S10)}$$

where n_γ and n_δ are the number of reactive DNA strands on a sphere and cube, respectively, and p_γ and p_δ are the bonding probabilities of the corresponding DNA types that are unhybridized.

The probabilities $p_{\gamma(\delta)}$ can be calculated using the following set of equations:

$$\begin{aligned} p_\gamma + N_s n_\delta p_\gamma p_\delta \exp(-\beta \Delta G_{\gamma\delta}) &= 1 \\ p_\delta + n_\gamma p_\gamma p_\delta \exp(-\beta \Delta G_{\gamma\delta}) &= 1 \end{aligned} \quad \text{(S11)}$$

where N_s is the number of spherical colloids. In the next section, we describe how we determined $n_{\gamma(\delta)}$, which is the other input parameter needed for the calculations.

Estimate of the density of DNA strands

Accurately estimating the number of strands that become embedded in the lipid bilayer solely based on the number of strands initially added to the system is challenging due to the uncertainty regarding the fraction of DNA that will successfully insert into the lipid layer. The experimentally employed nominal concentrations would correspond to one DNA linker per 50 nm^2 on the cubes and one inert DNA strand per 10 nm^2 on both particles if all strands are inserted. However, the hydrophobic cholesterol moeities of the linkers can also induce their assembly into small micellar-like clusters in solution. Thus, we expect there to be a balance between single DNA strands in solution, DNA strands in micelle-like clusters in

solution, and inserted into the colloid supported lipid bilayer. Hence, the actual number of strands available for binding is lower than the number of strands added in the experiments. Moreover, the total number of reactive strands is affected by the presence of inert strands that are added as stabilizers.

Therefore, we estimate the number of strands on the surface of spheres and cube using the predictions of our microscopic model for the free-energy barrier experienced by the smallest spheres we investigate, which have a size ratio of $\alpha = 0.63$. Experiments have shown that for this size ratio, motion around the cube is not hindered. This means that the free energy barrier for moving from one face to the other passing through an edge cannot be higher than $O(10^1)$ k_BT. If the barrier were higher, the configurational energy penalty for the strands would be too large, and the spheres would remain on the face of their initial adsorption.

To estimate the number of strands, we take several values of the total (reactive and inert) number of strands on the sphere, $n_\gamma^{tot} = n_\gamma + n_\gamma^{inert}$, and thus densities $\rho_\gamma^{tot} = n_\gamma^{tot}/A_s$ with A_s the surface area of the sphere, for the smallest size ratio α. We then calculate an approximate total number of strands on the cube, $n_\delta^{tot} = n_\delta + n_\delta^{inert}$, by fixing the surface density for both the spheres and the cube and using that the surface area of the cube in our case is $A_c = 4.9\sigma_c^2$. Here, we assume that, as a first approximation, there is no reason why the experimental procedure would give rise to an overall different density on the two colloid types. We then calculate the number of reactive and inert strands on each colloid, taking into account the density ratio between inert and reactive strands used in the experiments (see Methods). For spheres, this ratio is ≈ 330, while on the cube, the probability of having an inert strand is about five times higher than having a reactive one. We calculate βF_{bond} using Eq. S10 and perform Monte Carlo simulations based on the calculated free energy (see Methods). For each simulation, we calculate the autocorrelation function as defined in the main text and ensure that it decays within the simulation time. We present several examples in Figure S3 to show that for $\rho_\gamma = n_\gamma/A_s > 15$ (corresponding to a face-to-edge energy barrier higher than $\approx 11k_BT$), motion on the surface of the cube is completely

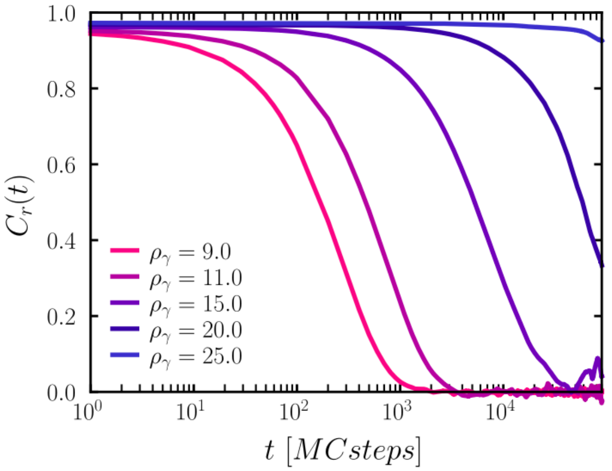

Figure S3: Position autocorrelation function $C_r(t)$ as a function of simulation time reported in MC steps for different strand densities in the sphere ρ_γ as indicated in the legend.

hindered, which would therefore contradict experimental evidence.

We choose a strand density of $\rho_\gamma = 11$ for the smallest investigated size ratio, which corresponds to a face-to-edge energy barrier of $\approx 7k_BT$ and results in an autocorrelation function that decays within 10^4 MC steps (see Figure S3). This value is only slightly different from other lower densities, and a different choice of ρ_γ in this range does not significantly affect the results. We also note that this density estimate is reasonable as it allows us to calculate the typical DNA width d based on $\rho = 1/(ld)$,[11] which gives a value of $d \approx 5$ nm, consistent with typical estimates for DNA width. Subsequently, we recompute $n_{\gamma(\delta)}$ for the higher size ratios while keeping the density of strands approximately fixed. The number of strands on the cube n_δ remains constant, as the cubes are the same for all size ratios. We have further verified that in all cases, the number of reactive strands present on the cube is sufficient for the reactive strands to form DNA patches on all spheres. Table S2 presents the parameters used for calculating the free energy for all the investigated size ratios.

Figure S4 illustrates the impact of varying $\rho_{\gamma(\delta)}$ on the change in free energy that the sphere experiences as it moves from the center of a face to an edge, i.e., $\beta\Delta F_{edge}$, for all size ratios. The left panel of Figure S4 shows the effect of varying the density of strands ρ_γ on a sphere, while keeping the density of reactive strands on the cube fixed so that $n_\delta = 3000$.

Table S2: Parameters employed for the calculation of the free energy βF_{bond} for the size ratios α investigated: n_γ number of strands in the sphere, n_γ^{inert} number of inert strands in the sphere, n_γ^{tot} total number of strands in the sphere, n_δ number of strands in the cube, n_δ^{inert} number of inert strands in the cube, n_δ^{tot} total number of strands in the cube, and the corresponding strand densities.

	Sphere			Cube		
α	n_γ	n_γ^{inert}	n_γ^{tot}	n_δ	n_δ^{inert}	n_δ^{tot}
0.63	14	4676	4690	3000	15000	18000
0.93	30	10020	10050	3000	15000	18000
1.20	50	16700	16750	3000	15000	18000
1.49	77	25718	25795	3000	15000	18000
1.98	136	45424	45560	3000	15000	18000
3.20	352	120908	121270	3000	15000	18000
4.44	682	227788	228470	3000	15000	18000
	Sphere			Cube		
	ρ_γ	ρ_γ^{inert}	ρ_γ^{tot}	ρ_δ	ρ_δ^{inert}	ρ_δ^{tot}
	≈ 11	≈ 3705	≈ 3716	≈ 613	≈ 3061	≈ 3674

The right panel, on the other hand, shows the opposite scenario, where n_γ is fixed to the values reported in Table S2. We note that choosing a larger value of n_γ would cause the free energy barrier to increase excessively, making it difficult to observe motion of the spheres.

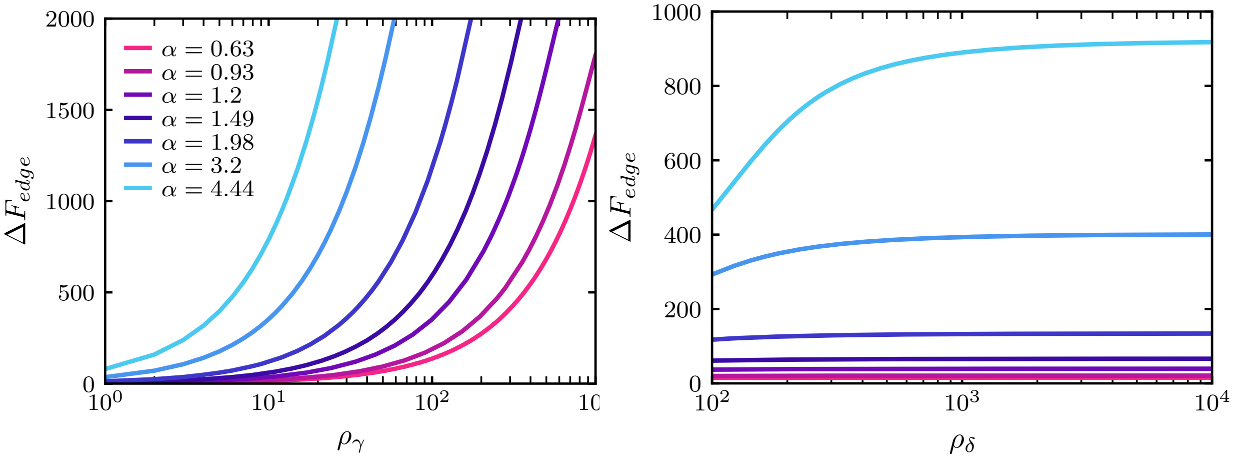

Figure S4: Free-energy difference $\beta\Delta F_{edge}$ between the center of a facet of the cube and a point on an edge (left) for varying density of strands on the sphere ρ_γ at fixed number of strands on the cube $n_\delta = 3000$ and (right) for varying density of the strands on the cube ρ_δ at a fixed number of strands on the spherical colloid for each size ratio (see Table S2).

On the contrary, increasing the number of strands on the cube would not result in any differences in the free energy barrier. This is consistent with our expectations, given that the number of strands on the spheres constitutes the upper limit for binding. Furthermore, this also supports the assumption of treating each sphere independently in the calculation of free energy, assuming $N_s = 1$. This is because the number of strands on the cube is sufficient to allow for bond formation for all the spheres at all size ratios.

Supplementary Figures and Tables

Table S3: Gravitational height of the silica particles used in the study.

Diameter of Sphere (μm)	Gravitational Height (nm)
0.66±0.01	2.8×10^3
0.97±0.05	8.8×10^2
1.25±0.05	4.1×10^2
1.55±0.05	2.2×10^2
2.06±0.05	92
3.32±0.05	22
4.62±0.05	8.1

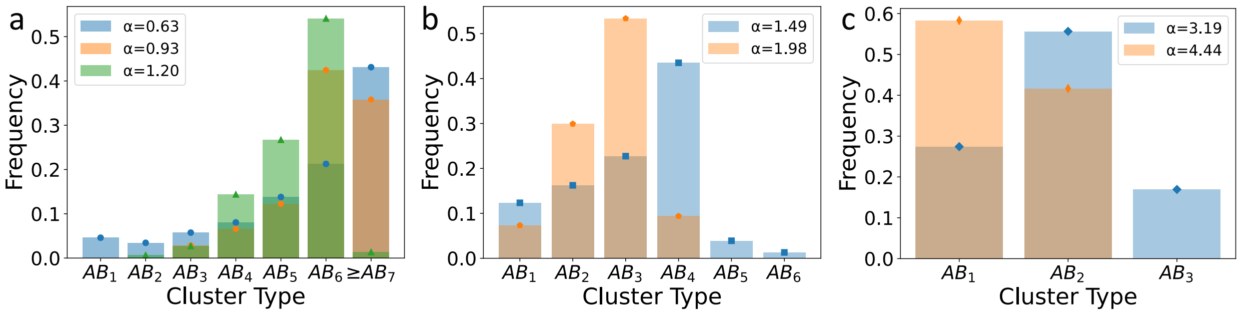

Figure S5: Cluster size distribution for varying sphere-to-cube size ratios a) α=0.63, 0.93 and 1.20. b) α=1.49 and 1.98, and c) α=3.19 and 4.44.

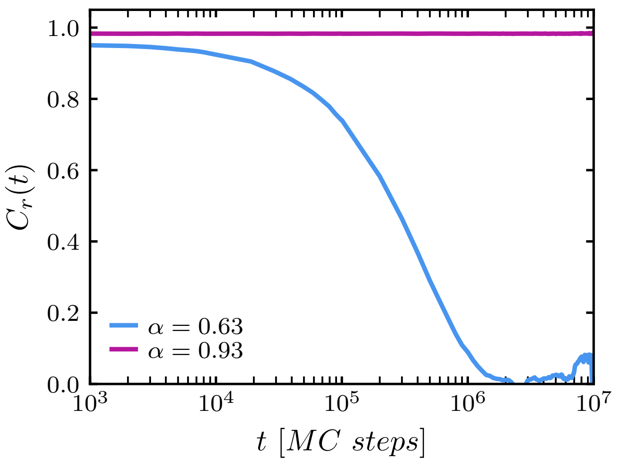

Figure S6: Position autocorrelation function for $\alpha = 0.63$ and 0.93 as a function of the simulations time reported as MC steps.

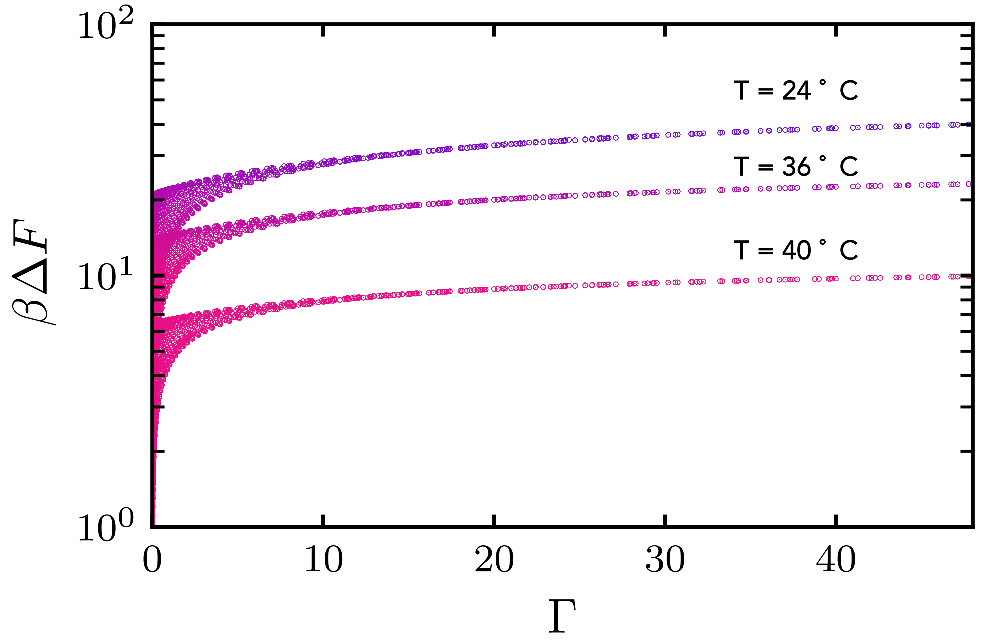

Figure S7: Free energy $\beta\Delta F$ as a function of the Gaussian curvature of the superball Γ for the three different temperatures analyzed, corresponding to $T = 24, 36, 40°\text{C}$.

Supplementary Movies

Movie S1: Confocal microscopy video of a flexible colloidal molecule consisting of 0.66 $\pm 0.01 \mu$m diameter spheres and a 1.04$\pm$0.04 μm cube, equivalent to a size ratio α =0.63. Video is shown in real time with a frame rate of 15.17 frames per second.

Movie S2: Bright-field microscopy video of a flexible colloidal molecule consisting of 0.97$\pm$0.05μm diameter spheres and a 1.04$\pm$0.04 μm cube, equivalent to a size ratio α = 0.93. Video is shown in real time with a frame rate of 20 frames per second.

Movie S3: Bright-field microscopy video of a flexible colloidal molecule consisting of 1.25 $\pm$0.05μm diameter spheres and a 1.04$\pm$0.04 μm cube, equivalent to a size ratio α = 1.20. Video is shown in real time with a frame rate of 25 frames per second.

Movie S4: Confocal microscopy video of a flexible colloidal molecule consisting of 1.55 $\pm$0.05μm diameter spheres and a 1.04$\pm$0.04 μm cube, equivalent to a size ratio α = 1.49. Video is shown in real time with a frame rate of 7.9 frames per second.

Movie S5: Bright-field microscopy video of a flexible colloidal molecule consisting of 2.06$\pm$0.05μm diameter spheres and a 1.04$\pm$0.04 μm cube, equivalent to a size ratio α = 1.98 Video is shown in real time with a frame rate of 18.3 frames per second.

Movie S6: Bright-field microscopy video of a flexible colloidal molecule consisting of 3.32 $\pm$0.05μm diameter spheres and a 1.04$\pm$0.04 μm cube, equivalent to a size ratio α = 3.19. Video is shown in real time with a frame rate of 18.5 frames per second.

Movie S7: Confocal microscopy video of a flexible colloidal molecule consisting of 4.62 $\pm$0.05μm diameter spheres and a 1.04$\pm$0.04 μm cube, equivalent to a size ratio α = 4.44. Video is shown in real time with a frame rate of 1.9 frames per second.

Movie S8: Bright-field microscopy video of a flexible colloidal molecule consisting of 0.66 $\pm$0.01μm diameter spheres and a 1.04$\pm$0.04 μm cube, equivalent to a size ratio α = 0.63. Video is shown in real time with a frame rate of 16.7 frames per second.

Movie S9: Bright-field microscopy video of a flexible colloidal molecule consisting of 0.97 $\pm$0.05μm diameter spheres and a 1.04$\pm$0.04 μm cube, equivalent to a size ratio α = 0.93.

Video is shown in real time with a frame rate of 10 frames per second.

Movie S10: Bright-field microscopy video of temperature switchable motion of a flexible colloidal molecule consisting of 0.97 $\pm 0.05 \mu$m diameter spheres and a 1.04$\pm$0.04 μm cube, equivalent to a size ratio $\alpha = 0.93$.

References

(1) Angioletti-Uberti, S.; Varilly, P.; Mognetti, B. M.; Frenkel, D. Mobile linkers on DNA-coated colloids: Valency without patches. *Physical review letters* **2014**, *113*, 128303.

(2) SantaLucia Jr, J. A unified view of polymer, dumbbell, and oligonucleotide DNA nearest-neighbor thermodynamics. *Proceedings of the National Academy of Sciences* **1998**, *95*, 1460–1465.

(3) Di Michele, L.; Mognetti, B. M.; Yanagishima, T.; Varilly, P.; Ruff, Z.; Frenkel, D.; Eiser, E. Effect of inert tails on the thermodynamics of DNA hybridization. *Journal of the American Chemical Society* **2014**, *136*, 6538–6541.

(4) Varilly, P.; Angioletti-Uberti, S.; Mognetti, B. M.; Frenkel, D. A general theory of DNA-mediated and other valence-limited colloidal interactions. *The Journal of chemical physics* **2012**, *137*, 094108.

(5) Parolini, L.; Mognetti, B. M.; Kotar, J.; Eiser, E.; Cicuta, P.; Di Michele, L. Volume and porosity thermal regulation in lipid mesophases by coupling mobile ligands to soft membranes. *Nature communications* **2015**, *6*, 1–10.

(6) Linne, C.; Visco, D.; Angioletti-Uberti, S.; Laan, L.; Kraft, D. J. Direct visualization of superselective colloid-surface binding mediated by multivalent interactions. *Proceedings of the National Academy of Sciences* **2021**, *118*, e2106036118.

(7) Mognetti, B. M.; Leunissen, M.; Frenkel, D. Controlling the temperature sensitivity of

DNA-mediated colloidal interactions through competing linkages. *Soft Matter* **2012**, *8*, 2213–2221.

(8) Geggier, S.; Kotlyar, A.; Vologodskii, A. Temperature dependence of DNA persistence length. *Nucleic acids research* **2011**, *39*, 1419–1426.

(9) González García, Á.; Opdam, J.; Tuinier, R. Phase behaviour of colloidal superballs mixed with non-adsorbing polymers. *The European Physical Journal E* **2018**, *41*, 1–15.

(10) Angioletti-Uberti, S.; Varilly, P.; Mognetti, B. M.; Tkachenko, A. V.; Frenkel, D. Communication: A simple analytical formula for the free energy of ligand–receptor-mediated interactions. *The Journal of chemical physics* **2013**, *138*, 01B401.

(11) McMullen, A.; Hilgenfeldt, S.; Brujic, J. DNA self-organization controls valence in programmable colloid design. *Proceedings of the National Academy of Sciences* **2021**, *118*, e2112604118.
